# The Association Between Pre-cycle Maternal Vitamin D Status and Reproductive Outcomes in Frozen-Thawed Embryo Transfer: A Systematic Review and Meta-Analysis

**DOI:** 10.7759/cureus.100977

**Published:** 2026-01-07

**Authors:** Muawia Ali Ahmed Abdelgader, Amani Abdallah Alhajj Abdallah, Khadija Alkahtani, Fetoon Ibrahim Ghanem, Fatimah Fuad Slais, Abrar Alsaeed, Kholoud Alraddadi, Ahmad Saleh Alnaim, Bara Mahdi Bahakeem, Fay Sharaf Althobaity

**Affiliations:** 1 Obstetrics and Gynecology, Al-Jouf Maternity and Children Hospital, Sakaka, SAU; 2 General Practice, Omdurman Islamic University, Omdurman, SDN; 3 Department of Obstetrics and Gynecology, Women's Health Center, King Abdullah Bin Abdulaziz University Hospital, Princess Nourah Bint Abdulrahman University, Riyadh, SAU; 4 General Practice, Faculty of Medical Sciences, University of Groningen, Groningen, NLD; 5 Surgery and Obstetrics, University of Galway, Galway, IRL; 6 Obstetrics and Gynecology, King Salman Bin Abdulaziz Medical City, Almedina, SAU; 7 Medicine, Faculty of Medical Sciences, University of Groningen, Groningen, NLD; 8 Medicine, College of Medicine, Jeddah University, Jeddah, SAU; 9 Medicine, King Abdulaziz University, Jeddah, SAU

**Keywords:** clinical pregnancy, frozen embryo transfer, infertility, live birth rate, meta-analysis, vitamin d

## Abstract

Vitamin D has been increasingly recognized for its potential role in reproductive physiology, particularly in endometrial receptivity and implantation. Despite growing interest, the impact of serum vitamin D status on outcomes of frozen embryo transfer (FET) cycles remains debated. The objective of this systematic review and meta-analysis was to assess the association between maternal vitamin D concentrations and reproductive success, specifically clinical pregnancy (CPR) and live birth rates (LBR), during frozen-thawed embryo transfer cycles, effectively focusing on the role of endometrial receptivity A systematic search was conducted in CENTRAL, Embase, MEDLINE, and Scopus for observational studies published until November 2025. Eligible studies compared reproductive outcomes in women with low versus normal serum 25-hydroxyvitamin D (25(OH)D) levels before autologous FET. The women were categorized as vitamin D insufficient if the level was <20 ng/ml and <30 ng/ml in different studies. The primary outcomes were the clinical pregnancy rate (CPR) and live birth rate (LBR). Data were pooled using a random-effects model, and the risk of bias was assessed using the Newcastle-Ottawa Scale (NOS). Four cohort studies comprising 2,061 FET cycles were included in the analysis. In the overall pooled analysis, low vitamin D levels were associated with lower odds of clinical pregnancy; however, this association did not reach statistical significance (OR: 0.37, 95% CI: 0.12-1.08), likely due to substantial heterogeneity among studies (I²=92.7%). When the analysis was restricted to high-quality research (involving 1,769 cycles), heterogeneity was eliminated (I²=0%), demonstrating a significant reduction in the likelihood of clinical pregnancy for patients with vitamin D deficiency (OR: 0.80, 95% CI: 0.65-0.99; p=0.03). Similarly, pooled analysis of live birth outcomes demonstrated a borderline reduction in live birth rates in the low vitamin D group, although this finding did not reach statistical significance (OR: 0.79, 95% CI: 0.60-1.04; p=0.08). Vitamin D deficiency appears to be associated with adverse reproductive outcomes in FET cycles, particularly when analyzing high-quality data. These findings suggest that low vitamin D levels are associated with adverse FET outcomes. However, as this review was limited to observational studies, these results identify deficiency as a prognostic marker rather than proving that screening and repletion directly optimize implantation and pregnancy success. Therefore, vitamin D sufficiency appears to be associated with poorer reproductive outcomes in (FET) cycles, but high-quality randomized trials are needed to establish definitive recommendations.

## Introduction and background

Infertility is a pervasive global health challenge, requiring the continuous refinement of assisted reproductive technologies (ART) to optimize clinical outcomes. Over the past decade, the ART has shifted toward the utilization of frozen-thawed embryo transfer (FET) cycles due to the advancements in cryopreservation techniques, specifically vitrification, which have mitigated cryodamage and improved post-thaw survival rates [1]. Furthermore, the adoption of "freeze-all" strategies has become prevalent to minimize the risk of ovarian hyperstimulation syndrome (OHSS) and circumvent the adverse effects of supraphysiologic hormonal milieus on endometrial receptivity observed during controlled ovarian stimulation [2,3]. As FET cycles decouple the ovarian stimulation phase from the implantation phase, the endometrium can be prepared using either natural ovulation or programmed regimens with exogenous estrogen and progesterone. This controlled environment eliminates the supraphysiologic hormonal levels seen in fresh cycles and makes it critical to identify modifiable prognostic factors that specifically influence endometrial receptivity and implantation success in the unique physiological context of a frozen cycle.

One such factor of increasing interest is vitamin D, a steroid hormone with a ubiquitous influence on human physiology. The presence of vitamin D receptors (VDR) and metabolic enzymes within key reproductive structures such as the placenta, endometrium, and ovaries underscores the hormone's essential role in reproductive physiology [4]. Low vitamin D levels are widespread among women of childbearing age and have been linked to the development of conditions associated with infertility, including endometriosis and polycystic ovary syndrome (PCOS) [4]. Biologically, the active form of vitamin D, 1,25-dihydroxyvitamin D3, regulates genes involved in implantation, cytokine production, and immune modulation at the maternal-fetal interface, suggesting a plausible mechanism by which serum levels could influence pregnancy outcomes.

The clinical association between vitamin D status and ART outcomes remains a subject of debate, as some investigators have reported a positive correlation between replete vitamin D levels and reproductive success, but others have found no significant association. A critical limitation of the literature is the heterogeneity of the study populations and treatment protocols. Most prior observational studies and meta-analyses have focused on fresh embryo transfers or pooled fresh and frozen cycles together [5-8]. In fresh cycles, the supraphysiologic estradiol levels induced by controlled ovarian stimulation can alter endometrial gene expression and induce premature maturation. This distinct, non-physiological environment may override or mask the subtle immunomodulatory benefits of vitamin D [3]. Therefore, extrapolating findings from fresh cycles to FET cycles may obscure the true relationship between vitamin D and implantation competence.

Furthermore, interventional studies investigating vitamin D supplementation often introduce heterogeneity by varying dosage, duration, and baseline characteristics, making it difficult to isolate the prognostic value of baseline vitamin D status itself [9]. Therefore, a distinct evaluation of FET cycles is necessary, as these cycles use artificial or natural endometrial preparation protocols that avoid the high gonadotropin and steroid levels characteristic of fresh stimulation [1], which creates a distinct endocrinological environment in which the impact of vitamin D on endometrial receptivity and early placentation may be more readily discernible.

To address this gap in the literature, this systematic review and meta-analysis aimed to evaluate the association between maternal serum vitamin D levels and reproductive outcomes, specifically in women undergoing FET. This review aims to provide a precise estimate of how baseline vitamin D status affects clinical pregnancy, live birth, and miscarriage rates in the specific context of FET, excluding fresh transfer cycles and active supplementation trials.

## Review

Methods

Protocol and Registration

This systematic review and meta-analysis were conducted in accordance with the Preferred Reporting Items for Systematic Reviews and Meta-Analyses (PRISMA) guidelines [10]. The study protocol was registered with the International Prospective Register of Systematic Reviews (PROSPERO) under the identification number CRD420251170965.

Search Strategy

An extensive search of key electronic databases such as Scopus, PubMed, MEDLINE, Embase, the Science Citation Index (SCI), and the Cochrane Central Register of Controlled Trials (CENTRAL) was conducted to identify relevant literature. The search window encompassed articles published until November 2025. The search strategy used a combination of Medical Subject Headings (MeSH) and free-text keywords relevant to the research question, including "vitamin D", "25-hydroxyvitamin D", "female infertility", "frozen embryo transfer", and "pregnancy outcome". No language restrictions were applied. The reference lists of all included studies and relevant reviews were manually screened ("snowballing") to identify additional eligible citations to ensure literature saturation.

Eligibility Criteria

The Population, Exposure, Comparator, Outcome, Study Design (PECOS) framework served as the guide for selecting eligible studies, as the population included women of reproductive age (≥18 years) undergoing autologous FET cycles using either programmed (artificial) or natural/modified endometrial preparation protocols. Maternal serum 25-hydroxyvitamin D (25(OH)D) concentrations were measured before the FET cycle. The primary comparison was between women categorized as vitamin D sufficient (normal) versus those with vitamin D deficiency or insufficiency (low). Classification was based on the cut-off values defined by the individual studies (typically <20 ng/mL or <30 ng/mL for the "low" group). The primary outcome, clinical pregnancy rate (CPR), was defined as the detection of a fetal heartbeat or gestational sac via ultrasound, alongside the live birth rate (LBR), defined as the delivery of a live infant ≥20 weeks of gestation. Study designs were restricted to observational cohort studies (prospective and retrospective) to ensure the temporal relationship between exposure (vitamin D measurement) and outcome (pregnancy).

Exclusion Criteria

To isolate the specific impact of baseline vitamin D status on FET outcomes, this review excluded: studies involving fresh embryo transfer cycles; cycles using donor gametes or gestational surrogacy; studies focusing on populations with specific comorbidities known to alter vitamin D metabolism (e.g., chronic renal failure); and interventional trials evaluating the therapeutic effect of high-dose vitamin D supplementation administered to correct deficiency during the study period. Conference abstracts, case reports, and reviews without original, extractable data were also excluded. Cross-sectional studies were excluded to minimize the risk of reverse causation.

Study Selection and Data Extraction

Records identified through database searches were imported into systematic review software, and duplicates were removed. Two independent authors screened the titles and abstracts for relevance. Full-text articles of potentially eligible studies were assessed against the inclusion/exclusion criteria. Disagreements were resolved by consensus between the two independent reviewers. 

Data extraction was performed independently by two authors using standardized forms. The extracted variables included study characteristics (author, year, country, design), participant demographics (age, BMI), infertility etiology, vitamin D measurement methods (e.g., enzyme-linked immunosorbent assay, ELISA; liquid chromatography-mass spectrometry, LC-MS), cut-off definitions, FET protocols, and raw numerical data for reproductive outcomes. For the meta-analysis, data for the vitamin D-deficient and insufficient groups were aggregated into a single "low vitamin D" category to be compared against the "replete" group. This aggregation combines distinct biological thresholds (<20 ng/mL and <30 ng/mL); however, this binary classification was necessary to maximize statistical power because of the limited number of eligible studies available for subgroup analysis.

Risk of Bias Assessment

The methodological quality of the included observational studies was evaluated using the Newcastle-Ottawa Scale (NOS) [11], which assesses three domains: the selection of study groups, the comparability of cohorts, and the ascertainment of exposure/outcomes. Studies were awarded stars, with a maximum score of nine. Studies scoring 7-9 were classified as high quality (low risk of bias), 4-6 as moderate quality, and 0-3 as low quality.

Statistical Analysis

R software (v. 4.5.1; R Foundation for Statistical Computing, Vienna, Austria), specifically the meta and metafor packages, was utilized for statistical evaluation. Odds ratios (ORs) and 95% confidence intervals (CIs) for every included study were computed to compare outcomes between patients with low vitamin D levels and those with normal vitamin D levels. Pooled effect estimates were generated using a random-effects model based on the DerSimonian-Laird method, accounting for both within-study and between-study variability. Statistical heterogeneity was quantified using the I^2 ^statistic, with values of 25%, 50%, and 75% representing low, moderate, and high heterogeneity, respectively. Sensitivity analyses were performed to assess the robustness of the findings by restricting the analysis to high-quality studies (low risk of bias). Publication bias assessment via funnel plots and Egger's test was planned for outcomes including ten or more studies. A two-sided p-value <0.05 was considered statistically significant.

Results

Search Results and Study Selection

**Figure 1:**
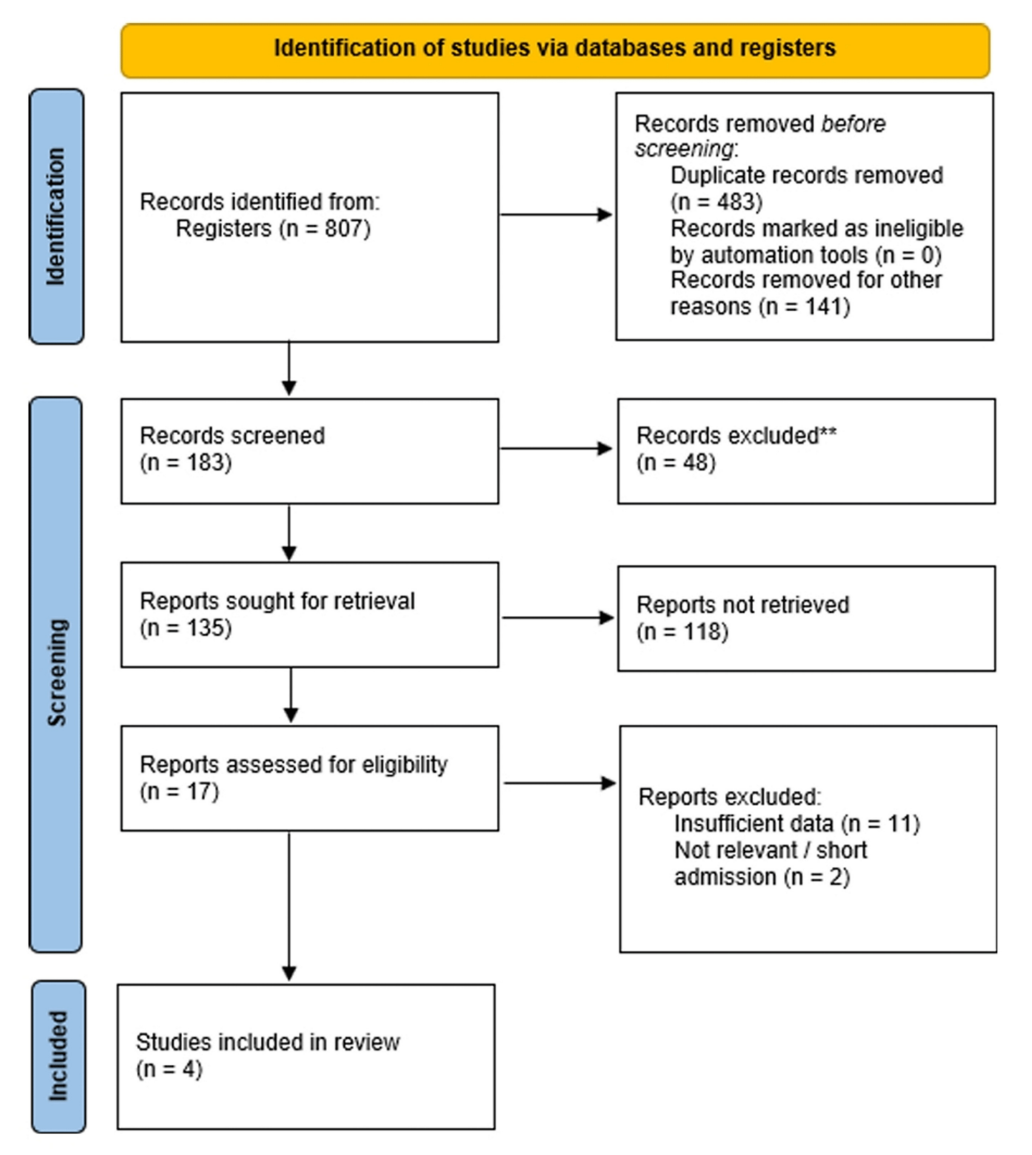
PRISMA flow diagram

The literature search across electronic databases yielded 807 records screened. After removing 483 duplicates, 324 unique citations were screened based on their titles and abstracts. Of these, 135 full-text articles were assessed for eligibility. Ultimately, four studies met the inclusion criteria and were included in the quantitative meta-analysis [12-15]. The study selection process is illustrated in the PRISMA flowchart (Figure 1).

Study Characteristics

The included studies were published between 2016 and 2025 and comprised 2,061 FET cycles. The study designs included two prospective cohort studies [12,15] and two retrospective cohort studies [13,14]. The studies were conducted in Egypt [12,15], Hong Kong [13], and Belgium [14]. Vitamin D levels were assessed using enzyme-linked immunosorbent assay (ELISA) or automated immunoassays in three studies, whereas Ko et al. [13] used liquid chromatography-mass spectrometry (LC-MS). The threshold for defining vitamin D deficiency/insufficiency (low vitamin D) varied, with two studies using a cut-off of <30 ng/mL [12,15] and two studies using <20 ng/mL [13,14]. Data regarding statistical adjustment were also extracted as the two high-quality studies [13,14] performed multivariable analyses adjusting for critical confounders such as age, BMI, and season, whereas the remaining studies [12,15] reported unadjusted outcomes. The characteristics of the included studies are summarized in Table 1.

**Table 1:** Characteristics of included studies FET - frozen-thawed embryo transfer; ICSI - intracytoplasmic sperm injection; ELISA - enzyme-linked immunosorbent assay; LC-MS - liquid chromatography-mass spectrometry; AMH - anti-Müllerian hormone

Study	Year	Country	Study design	Sample size (n)	Population	Vitamin D assay method	Adjustments	Cut-off (low vitamin D)
Ahmed et al. [12]	2024	Egypt	Prospective cohort	188	Infertile women undergoing ICSI/FET	Enzyme immunoassay	None (univariate)	<30 ng/mL
Ko et al. [13]	2025	Hong Kong	Retrospective cohort	1489	Women undergoing FET	LC-MS	Age, BMI, AMH, protocol	<20 ng/mL
van de Vijver et al. [14]	2016	Belgium	Prospective cohort	280	Women undergoing FET	Automated immunoassay	Age, BMI, season, ethnicity, smoking	<20 ng/mL
Bendary et al. [15]	2023	Egypt	Prospective cohort	104	Infertile women undergoing ICSI/FET	ELISA	None (univariate)	<30 ng/mL

Risk of Bias Assessment

**Figure 2:**
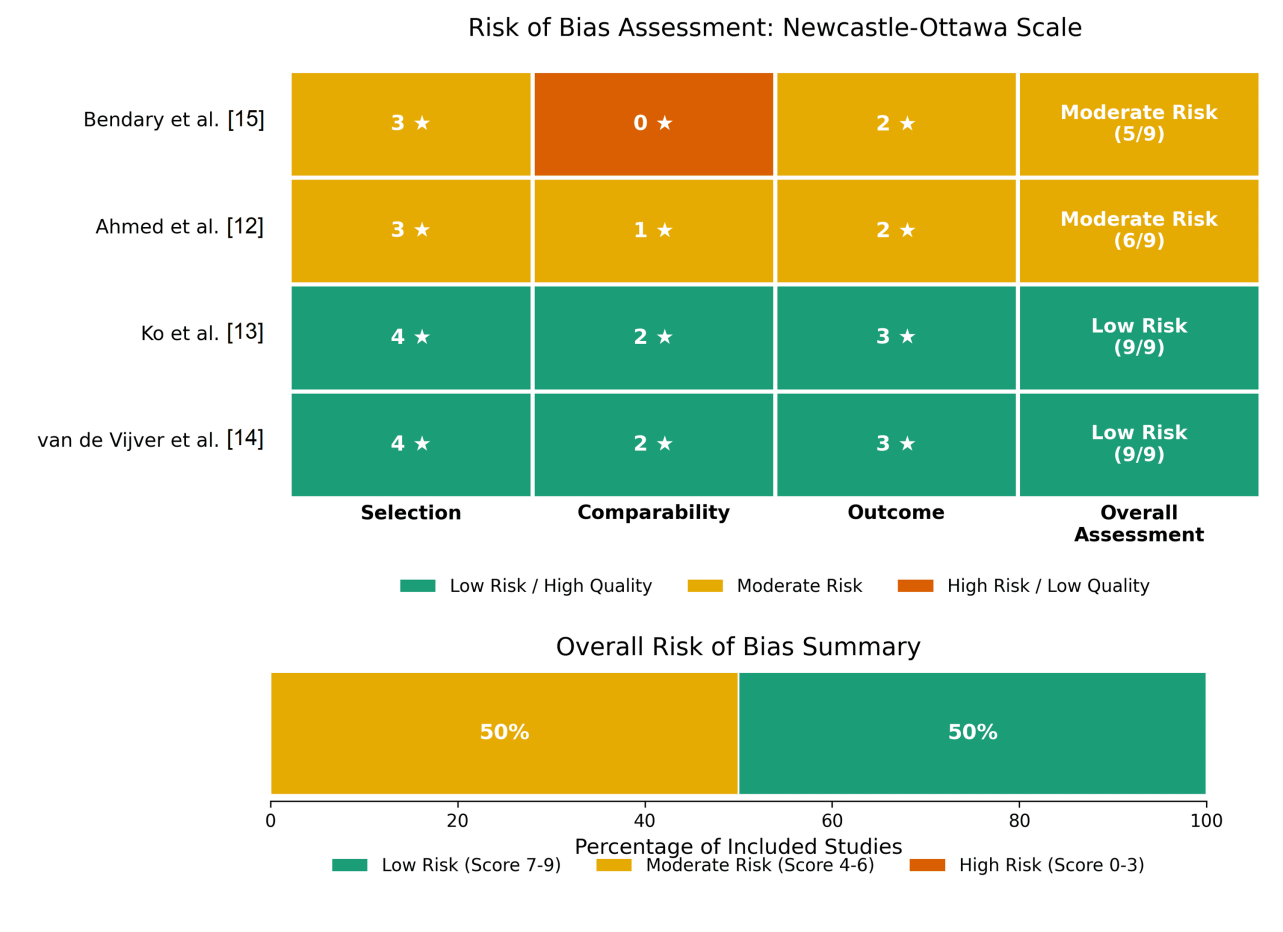
Risk of bias assessment using the Newcastle-Ottawa Scale

Study quality was evaluated by the NOS, as two studies [13,14] received a maximum 9-star rating, indicating high quality and a low risk of bias. The remaining two studies [12,15] were classified as moderate quality, primarily due to limitations in the comparability domain, specifically the lack of robust multivariable adjustment for confounders. A summary of the risk of bias assessment is presented in Figure 2.

Meta-Analysis of Clinical Pregnancy Rate

**Figure 3:**
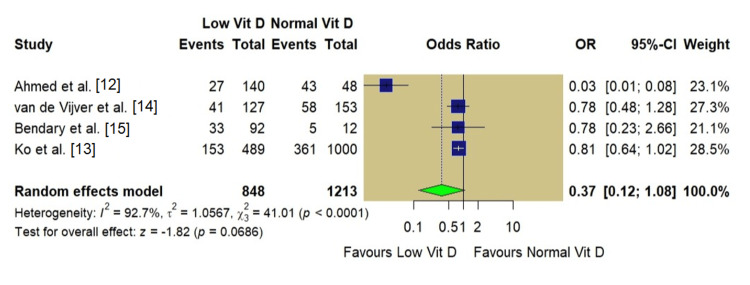
Forest plot of clinical pregnancy rate (low vs. normal vitamin D)

All four included studies reported CPR as an outcome. The pooled analysis comprised 2,061 women, including 848 with low vitamin D levels and 1,213 with normal vitamin D levels. In the pooled analysis based on a random-effects model, women with low vitamin D levels had lower odds of achieving clinical pregnancy compared with those with normal levels; however, this association did not reach statistical significance (OR: 0.37, 95% CI: 0.12-1.08; p=0.06). Substantial heterogeneity was observed across studies (I²=92.7%, p<0.001; Figure 3).

Sensitivity Analysis of Clinical Pregnancy Rate

**Figure 4:**
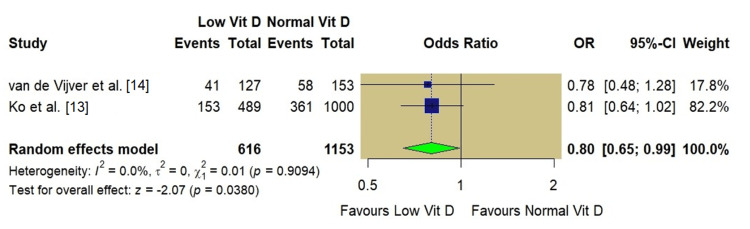
A sensitivity analysis of the clinical pregnancy rate restricted to high-quality studies

A sensitivity analysis was restricted to high-quality studies [13,14] to address the high heterogeneity observed in the CPR analysis and the impact of study quality. In this subgroup, the association between low vitamin D and clinical pregnancy became more precise, showing a trend toward lower pregnancy rates in the low vitamin D group (OR: 0.80, 95% CI: 0.65-0.99; p=0.03), with heterogeneity completely resolved (I^2^=0%; Figure 4).

Publication Bias of Clinical Pregnancy Rate

**Figure 5:**
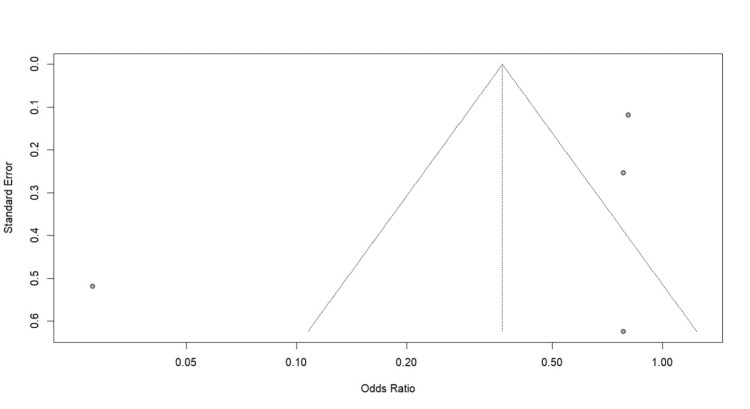
Funnel plot of clinical pregnancy rate

Visual inspection of the funnel plot for clinical pregnancy rate (Figure 5) suggested potential asymmetry; however, formal statistical testing for publication bias (Egger's test) was not performed because of the limited number of included studies (n<10).

Meta-Analysis of Live Birth Rate

**Figure 6:**
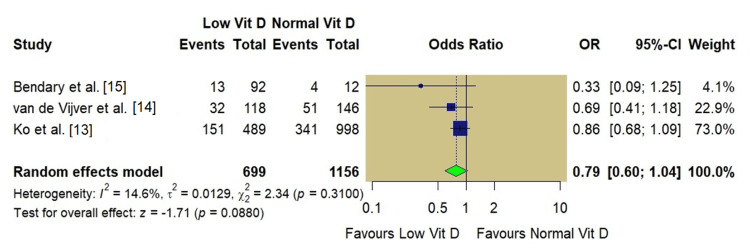
Forest plot of live birth rate (low vs. normal vitamin D)

LBR was reported in three studies [13-15]. The pooled analysis comprised 699 women in the low vitamin D group and 1,156 women in the normal vitamin D group. The random-effects model demonstrated no statistically significant association between vitamin D status and live birth rate (OR: 0.79, 95% CI: 0.60-1.04; p=0.08). The heterogeneity for this outcome was low (I^2^=14.6%, p=0.31). The forest plot for live birth rate is seen in Figure 6.

Discussion

Principal Findings and Interpretation

This study offers a targeted assessment of how maternal vitamin D status influences reproductive outcomes, focusing specifically on FET cycles to eliminate the confounding supraphysiologic hormonal milieu characteristic of fresh embryo transfers. The primary analysis revealed a general trend toward lower clinical pregnancy and live birth rates in women with vitamin D deficiency or insufficiency. However, the overall pooled analysis for CPR was limited by significant statistical heterogeneity (I^2^=92.6%). This heterogeneity appears to be driven by the magnitude of the effect reported in moderate-quality studies [12,15], which observed more drastic reductions in pregnancy rates compared to high-quality cohorts [13,14].

This discrepancy stems from methodological variations as the studies contributing to high heterogeneity utilized ELISA assays and a broader definition of deficiency (<30 ng/mL), while the sensitivity analysis of high-quality studies, where observed statistical heterogeneity was absent (I^2^=0%), comprised studies using more robust assay methods (LC-MS or automated immunoassay) and a stricter diagnostic threshold (<20 ng/mL), however, that heterogeneity estimates (I^2^) can be imprecise in small subgroups, so this finding should be interpreted as an indication of greater consistency rather than absolute homogeneity.

When the analysis was restricted to these high-quality studies with low risk of bias, rigorous multivariable adjustments, and precise exposure ascertainment, a statistically significant reduction in the odds of clinical pregnancy (OR: 0.80) was observed for women with low vitamin D. Similarly, the analysis of LBR showed a borderline trend (p=0.08) with low heterogeneity. These findings suggest a modest and consistent negative association between hypovitaminosis D and implantation success, aligning with the biological understanding that while vitamin D is not the sole determinant of implantation, it acts as a significant modulator of endometrial competence.

Biological Plausibility in the Context of FET

The restriction of this review to FET cycles allows for a focused examination of vitamin D's role in endometrial receptivity, independent of ovarian stimulation parameters. Biologically, vitamin D receptors (VDRs) are upregulated in the human endometrium during the window of implantation, and the active form, 1,25(OH)2D3, modulates HOXA10 expression, a gene essential for the implantation process [4]. Also, vitamin D possesses potent immunomodulatory properties, shifting the maternal immune response from a proinflammatory Th1 cytokine profile to a tolerogenic Th2 profile, which is essential for trophoblast invasion and early placentation [6]. In fresh in vitro fertilization (IVF) cycles, supraphysiologic estradiol levels may alter endometrial gene expression, masking the subtle immunomodulatory benefits of vitamin D. By using FET cycles, where the hormonal environment is more physiological, the findings reinforce the hypothesis that systemic vitamin D sufficiency is a prerequisite for optimal endometrial receptivity and placentation.

Strengths and Limitations

A major strength of this review is its strict eligibility criteria, which excluded fresh transfers and interventional supplementation trials, reducing heterogeneity related to the ovarian stimulation response and variable dosing regimens. Also, the use of the NOS allowed for a nuanced sensitivity analysis that clarified the impact of study quality on pooled effect estimates.

However, the number of eligible studies was small (k=4), limiting the power to detect smaller effect sizes or formally assess publication bias. Visual inspection of the funnel plot suggested asymmetry, indicating potential publication bias. Studies reporting a positive association are more likely to be published, whereas smaller studies finding no effect may remain unpublished (the 'file-drawer problem'). The pooled effect estimates may overestimate the true impact of vitamin D deficiency. While the aggregated sample size was substantial (n=2,061), the low number of included studies limits statistical power. The analysis may be underpowered to confirm smaller but clinically meaningful differences, particularly for the LBR, where a borderline non-significant trend (p=0.08) was observed. Additional data are needed to determine if this result represents a true negative or a type II error. Additionally, standard random-effects models may underestimate the error in small meta-analyses (small-study effects), further warranting a conservative interpretation of the pooled estimates.

Also, there was variability in the definition of low vitamin D, with cut-offs ranging from 20 ng/mL to 30 ng/mL. Although these thresholds are clinically accepted, this inconsistency complicates the determination of a precise threshold below which reproductive harm occurs. Moreover, this heterogeneity forces a binary risk model (low vs. normal) that obscures potential dose-response relationships. Women with severe deficiency (e.g., <10-15 ng/mL) behave biologically differently than those with borderline insufficiency (<20-30 ng/mL). Consequently, this aggregation assumes linearity without justification and limits the ability to determine if the observed adverse outcomes are driven specifically by severe deficiency. The small number of eligible studies (k=4) precluded a stratified analysis to investigate this distinction.

As with all observational meta-analyses, unmeasured confounding (e.g., lifestyle factors, sun exposure, race/ethnicity) cannot be fully ruled out, although high-quality studies adjusted for major confounders such as BMI and age. Finally, most data were obtained from specific geographic regions (Egypt, Hong Kong, and Belgium), which restricts generalizability, as vitamin D baseline levels and metabolism vary significantly by latitude, skin pigmentation (ethnicity), and cultural practices regarding sun exposure. Therefore, these findings may not be fully extrapolatable to populations in different geographic settings or with different cultural norms. Finally, regarding the study protocol, the PROSPERO registration (CRD420251170965) was completed after the initial literature search had commenced.

Clinical Implications and Future Directions

The findings of this meta-analysis have clinical relevance because of the low cost and high safety profile of vitamin D supplementation and the potential association with improved FET outcomes shown in our high-quality sensitivity analysis; therefore, vitamin D status appears to be a relevant prognostic factor in FET cycles. However, as the current evidence is primarily observational, association does not prove causality. Because of the potential for unmeasured confounding, the fact that supplementation trials were excluded from this analysis, and that the live birth rate analysis did not reach statistical significance (p=0.08), universal screening and repletion cannot be firmly recommended as standard clinical practice until these findings are confirmed by randomized interventional data. Future research should prioritize large-scale randomized control trials (RCTs), specifically within FET populations, to establish causality, as these trials should use standardized assay methods (such as LC-MS), uniform deficiency cut-offs, and investigate whether specific subgroups (e.g., women with PCOS or recurrent implantation failure) derive greater benefits from repletion.

## Conclusions

The findings indicate that women undergoing FET who are vitamin D-deficient may face significantly poorer reproductive outcomes. The overall evidence is heterogeneous, but data from high-quality studies indicate that women with low vitamin D levels have significantly reduced odds of clinical pregnancy and a trend toward lower live birth rates. Ensuring vitamin D sufficiency before FET represents a potential therapeutic target, though randomized trials are necessary to confirm if supplementation improves success rates in assisted reproduction.
